# *Magnolia kobus* DC. Alleviates adenine-induced chronic kidney disease by regulating ferroptosis in C57BL/6 mice

**DOI:** 10.3389/fphar.2025.1548660

**Published:** 2025-04-29

**Authors:** Jong Min Kim, Yiseul Kim, Hyun-Jin Na, Haeng Jeon Hur, Sang-Hee Lee, Mi Jeong Sung

**Affiliations:** Aging and Metabolism Research Group, Korea Food Research Institute, Wanju-gun, Republic of Korea

**Keywords:** *Magnolia kobus* DC., chronic kidney disease, ferroptosis, fibrosis, ferritinophagy

## Abstract

*Magnolia kobus* DC. (MO) is a medicinal plant that reportedly possesses various bioactive properties, including anti-hyperplastic, anti-inflammatory, and anti-cancer effects. Chronic kidney disease (CKD) is a progressive disorder characterized by inflammation, fibrosis, and oxidative stress, which leads to renal dysfunction. This study aimed to evaluate the renoprotective effects of MO against adenine-induced CKD in C57BL/6 mice. MO significantly attenuated renal injury by reducing blood urea nitrogen level and morphological change. Additionally, MO effectively reduced inflammation by inhibiting the expression of tumor necrosis factor-α, interleukin (IL)-1β, IL-6, monocyte chemoattractant protein-1, F4/80, intercellular adhesion molecule-1, and vascular cell adhesion molecule-1. MO also considerably ameliorated adenine-induced renal fibrosis by regulating the suppressor of mothers against decapentaplegic/matrix metalloproteinase signaling. Furthermore, MO significantly protected against renal senescence by reducing the protein expression of p53, p16, and p21 induced by CKD. Additionally, MO supplementation suppressed CKD-induced ferroptosis and ferritinophagy by regulating the protein expression of SLC7A11 glutathione peroxidase 4, prostaglandin-endoperoxide synthase 2, human palmitoyl-CoA ligase, NADPH oxidase 4, 4-hydroxynonenal, transferrin receptor, heme oxygenase-1, nuclear receptor coactivator 4, beclin-1, microtubule-associated proteins 1A/1B light chain 3B, and kallikrein-related peptidase 4. In conclusion, this study suggests that MO may be a potential functional food, pharmaceutical, or medicinal plant that can help regulate mechanisms associated with renal health.

## 1 Introduction

The prevalence of chronic kidney disease (CKD) is increasing globally, causing serious social and economic problems (Schieppati and Remuzzi, 2005). While patients with CKD exhibit minimal symptoms during the early stages, CKD leads to various complications and considerably reduces patients’ quality of life as their renal function gradually worsens (Webster et al., 2017). Furthermore, CKD requires expensive treatments and long-term management, including kidney transplantation, resulting in exceedingly high medical expenses (Parajuli et al., 2016). CKD is mainly caused by complex interactions among various physiological and cellular mechanisms (Gajjala et al., 2015). Renal fibrosis is a leading cause of CKD progression, and renal function is gradually impaired following the accumulation of abnormal extracellular matrix (ECM) (Bülow and Boor, 2019). Additionally, cellular senescence leads to inflammatory responses by continuously damaging the renal tissues and impeding functional recovery (Valentijn et al., 2018). Ferroptosis, the process of cell death caused by abnormal iron accumulation, has recently been shown to be an important factor contributing to renal damage and disease progression (Zhang and Li, 2022). When ferritinophagy is not regulated in the pathological state, it aggravates damage to kidney cells and accelerates CKD progression (Zhang and Li, 2022). These interacting mechanisms affect the onset and progression of CKD, ultimately increasing the risk of cardiovascular disease and mortality (Podkowińska and Formanowicz, 2020). Thus, the intake of functional foods with excellent physiological activities is important (Pang et al., 2012), given that functional foods with anti-inflammatory and antioxidative effects can help in slowing down or preventing CKD progression (Mafra et al., 2021). Therefore, the active intake of such functional foods is recommended to reduce the risk of CKD and maintain renal health.

*Magnolia kobus* DC. (MO) is a medicinal plant officially registered in the Asian National Pharmacopoeia. MO is produced by drying the flower buds of MO trees, which are primarily found in East Asia (Shen et al., 2008). MO exhibits physiological, anti-obesity, and anti-inflammatory effects and contains various bioactive compounds such as terpenoids, lignans, and neolignans (Kong et al., 2011; Lee et al., 2023; Shen et al., 2008). While various studies have demonstrated a correlation between CKD and ferroptosis, only a few studies have investigated the preventive effects of MO. Therefore, the current study aimed to investigate the protective effects of MO against renal damage, including fibrosis, senescence, ferroptosis, inflammation, and ferritinophagy, in mice with adenine-induced CKD.

## 2 Methods

### 2.1 Sample preparation

MO cultivated in Jeollabuk-do, Republic of Korea, was ground into a powder and extracted with a 50-fold volume of 50% ethanol at 40°C for 24 h. The extracted samples were filtered through a Whatman No. 2 filter paper (Whatman International Ltd., Kent, UK) and concentrated using a vacuum rotary evaporator. These samples were lyophilized and stored at −20°C until use.

### 2.2 Animal experimental design

Six-week-old C57BL/6 male mice were purchased from Oriental Biotechnology (Daejeon, Republic of Korea). The experimental animals were adapted at standard temperature (22°C ± 2°C) and humidity (50% ± 60%) on a 12/12 h light/dark cycle with free access to fodder and water. The experimental groups were randomly assigned to a normal chow diet group with vehicle (NC group, *n* = 10), adenine diet-induced CKD group (Ade group, *n* = 10), or adenine diet with 100 mg/kg/day MO group (Ade + MO group, *n* = 10). The adenine diet (0.25% w/w) was administered for 4 weeks to induce CKD. MO was administered by oral gavage for 4 weeks. At the end of the 4-week experimental period, mice were euthanized using 2.5% isoflurane anesthesia, and blood samples were collected immediately from the inferior vena cava. Renal tissues were fixed with 4% formaldehyde for histopathological analysis. Animal experimental procedures were approved by the Institutional Animal Care and Use Committee (IACUC) of the Korea Food Research Institute (certificate number: KFRI-M-22005) and were performed in accordance with the institutional guidelines established by the ethics committee.

### 2.3 Serum analysis

Serum biochemicals such as blood urea nitrogen (BUN), creatinine, calcium, and phosphorus were measured using a AU480 chemistry analyzer (Beckman Coulter, Brea, CA, United States) according to the manufacturer’s instructions.

### 2.4 Histopathological analysis

Fixed renal tissues were dehydrated, embedded in paraffin, sectioned at 4-μm thickness, dewaxed, and rehydrated. Sliced organs were stained with hematoxylin and eosin (H&E), periodic acid-Schiff, Masson’s trichrome, and immunohistochemistry. Stained samples were analyzed under a panoramic microscope (Olympus Corporation, Tokyo, Japan). For immunohistochemistry staining, the sliced tissues were incubated with anti-alpha-smooth muscle actin (anti-αSMA) antibodies (ab5694, 1:100, Abcam, Cambridge, UK) at 4°C overnight. After incubation with secondary antibodies, the immunohistochemistry scores were evaluated and visualized with 3,3′-diaminobenzidine (Dako, Glostrup, Denmark) using ImageJ software (NIH, Bethesda, ME, United States). Images were analyzed using CaseViewer (3DHISTECH Ltd., Budapest, Hungary).

### 2.5 Western blot analysis

Kidney proteins were extracted using a lysis buffer (9803S; Cell Signaling Technology, Danvers, MA, United States). The extracted proteins were separated using sodium dodecyl sulfate-polyacrylamide gel electrophoresis and transferred onto polyvinylidene fluoride membranes. The separated proteins were incubated with 3% bovine serum albumin in Tris-buffered saline and 0.1% Tween-20 at 25°C for 1 h. The incubated membranes were reacted with the following primary antibodies overnight at 4°C: intercellular adhesion molecule-1 (ICAM1) (ab109361, Abcam), vascular cell adhesion molecule-1 (VCAM1) (ab174279, Abcam), tumor necrosis factor-α (TNF-α), αSMA (ab14106, Abcam), matrix metalloproteinase (MMP)-9 (ab283575, Abcam), MMP-2 (ab92536, Abcam), suppressor of mothers against decapentaplegic (Smad) 2/Smad3 (3102S, Cell Signaling Technology), p53 (sc-126, Santa Cruz Biotechnology, Dallas, TX, United States), p16 (sc-166760, Santa Cruz Biotechnology), p21 (sc-6246, Santa Cruz Biotechnology), SLC7A11 (xCT) (ab175186, Abcam), glutathione peroxidase 4 (GPX4) (ab125066, Abcam), prostaglandin-endoperoxide synthase 2 (Ptgs2) (ab15191, Abcam), 4-hydroxynonenal (4-HNE) (ab46545, Abcam), transferrin receptor (TFR) (ab269513, Abcam), heme oxygenase-1 (HO-1) (10,701-1-ap, Proteintech, Rosemont, IL, United States), nuclear receptor coactivator 4 (NCOA4) (ab314553, Abcam), Beclin-1 (ab210498, Abcam), microtubule-associated proteins 1A/1B light chain 3B (LC3B 1/2) (ab48394, Abcam), and kallikrein-related peptidase 4 (KLF4) (ab106629, Abcam) with β-actin (sc-47778, Santa Cruz Biotechnology) being used as an internal control. Membranes were incubated with secondary antibodies (Cell Signaling Technology). The luminescence of each band was detected using ChemiDoc (Bio-Rad, Hercules, CA, United States). Protein density was estimated using ImageJ software (NIH).

### 2.6 Quantitative real-time PCR (qRT-PCR)

RNA was isolated from the renal tissues using an RNeasy RNA isolation kit (Qiagen, Valencia, CA, United States). The RNA concentration was measured using a NanoDrop spectrophotometer (Thermo Fisher Scientific, Waltham, MA, United States). The extracted RNA was reverse-transcribed into cDNA, and the synthesized cDNA was used for qRT-PCR using the SYBR Green PCR Master Mix Kit (Bio-Rad). The following primer sequences (5′–3′) were used in this study: *TNF-α* forward (F), TCT TCT CAT TCC TGC TTG TGG; *TNF-α* reverse (R), GGT CTG GGC CAT AGA ACT GA; *interleukin (IL)-1β* F, TGA GCT CGC CAG TGA AAT GAT; *IL-1β* R, TCC ATG GCC ACA ACA ACT GA; *IL-6* F, TGA GAG TAG TGA GGA ACA AG; *IL-6* R, CGC AGA ATG AGA TGA GTT G; *monocyte chemoattractant protein-1 (MCP-1)* F, GCC TGC TGT TCA CAG TTG C; *MCP-1* R, CAG GTG AGT GGG GCG TTA; *F4/80* F, CCT GGA CGA ATC CTG TGA AG; *F4/80* R, GGT GGG ACC ACA GAG AGT TG; *Col1A1* F, GAA CGC GTG TCA TCC CTT GT; *Col1A1* R, GAA CGA GGT AGT CTT TCA GCA ACA; *type IV collagen* F, TTA AAG GAC TCC AGG GAC CAC; *type IV collagen* R, CCC ACT GAG CCT GTC ACA C; *fibronectin* F, CCC TAT CTC TGA TAC CGT TGT CC; *fibronectin* R, TGC CGC AAC TAC TGT GAT TCG G; *TGFβ-1* F, TCA GAC ATT CGG GAA GCA GT; *TGFβ-1* R, ACG CCA GGA ATT GTT GCT AT; *glyceraldehyde 3-phosphate dehydrogenase (GAPDH)* F, ATT GTC AGC AAT GCA TCC TG; and *GAPDH* R, ATG GAC TGT GGT CAT GAG CC, with GAPDH serving as an internal control.

### 2.7 Statistical analysis

The experimental results are presented as mean ± standard error of the mean. Data normality was assessed using the Shapiro-Wilk test. Parametric statistical analyses were performed using one-way analysis of variance (ANOVA), followed by Tukey’s post-hoc test for multiple group comparisons. Using GraphPad Prism software version 10 (La Jolla, CA, United States). Statistical significance was set at *P* < 0.05, 0.01, 0.001, and 0.0001.

## 3 Results

### 3.1 Changes in body weight and serum biochemicals

**FIGURE 1:**
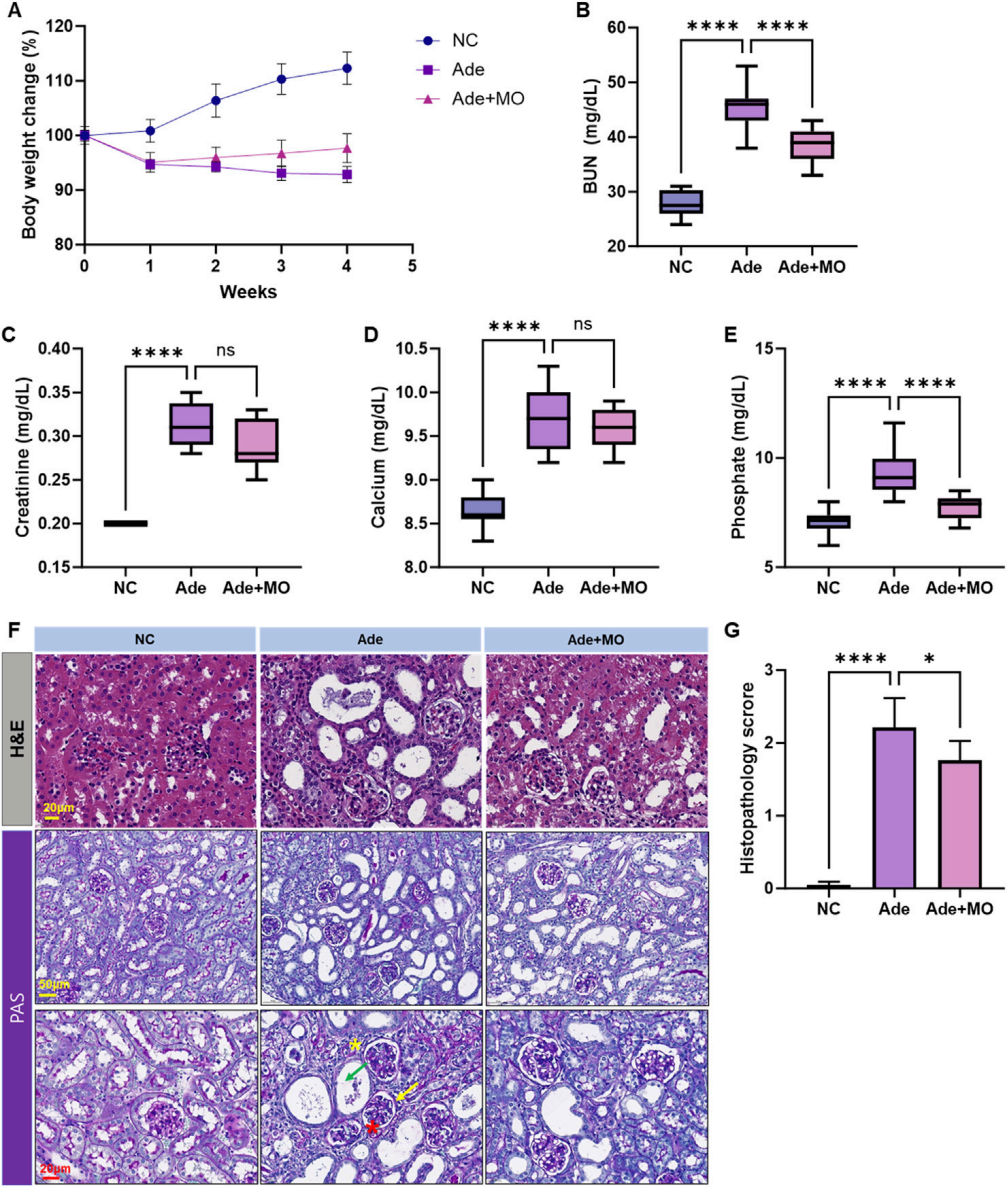
MO suppresses adenine-induced changes in body weight and serum biochemicals. Changes in **(A)** body weight change, **(B)** blood urea nitrogen (BUN) in serum, **(C)** creatinine in serum, **(D)** calcium levels in serum, **(E)** phosphorous levels in serum, **(F)** Upper panel: kidney stained with H&E. Lower panel: kidney stained with periodic acid-Schiff, **(G)** Histopathology score. Results are presented as mean ± standard error of the mean. The statistical significance indicated as *P < 0.05, **P < 0.01, ***P < 0.001, and ****P < 0.001, and ns means no significance.

The experimental mice were fed an adenine diet for 4 weeks to create a CKD model. The body weight of mice in the NC group continuously increased during the experimental period; conversely, the body weight of mice in the Ade group significantly decreased. Body weight reduction was ameliorated through MO intake, as compared with that in the Ade group (Figure 1A). Serum BUN, creatinine, calcium, and phosphorous levels increased, as compared with those in the NC group (Figures 1B–E). MO intake reduced the levels of certain serum biochemicals; in particular, serum BUN and phosphorus levels were significantly ameliorated. H&E and periodic acid-Schiff (PAS) staining could effectively evaluate the pathological changes in CKD, including inflammation, and fibrosis in CKD models. Thus, H&E and periodic acid-Schiff staining of the kidneys was performed to confirm the protective effects of MO against histopathological changes (Figures 1F,G). Renal damage, including inflammatory cell infiltration, renal tubular dilation, and atrophic basal membranes, was observed in the Ade group. However, MO administration significantly prevented renal damage, as compared with that in the Ade group.

### 3.2 Inflammatory activity in the renal tissues

**FIGURE 2:**
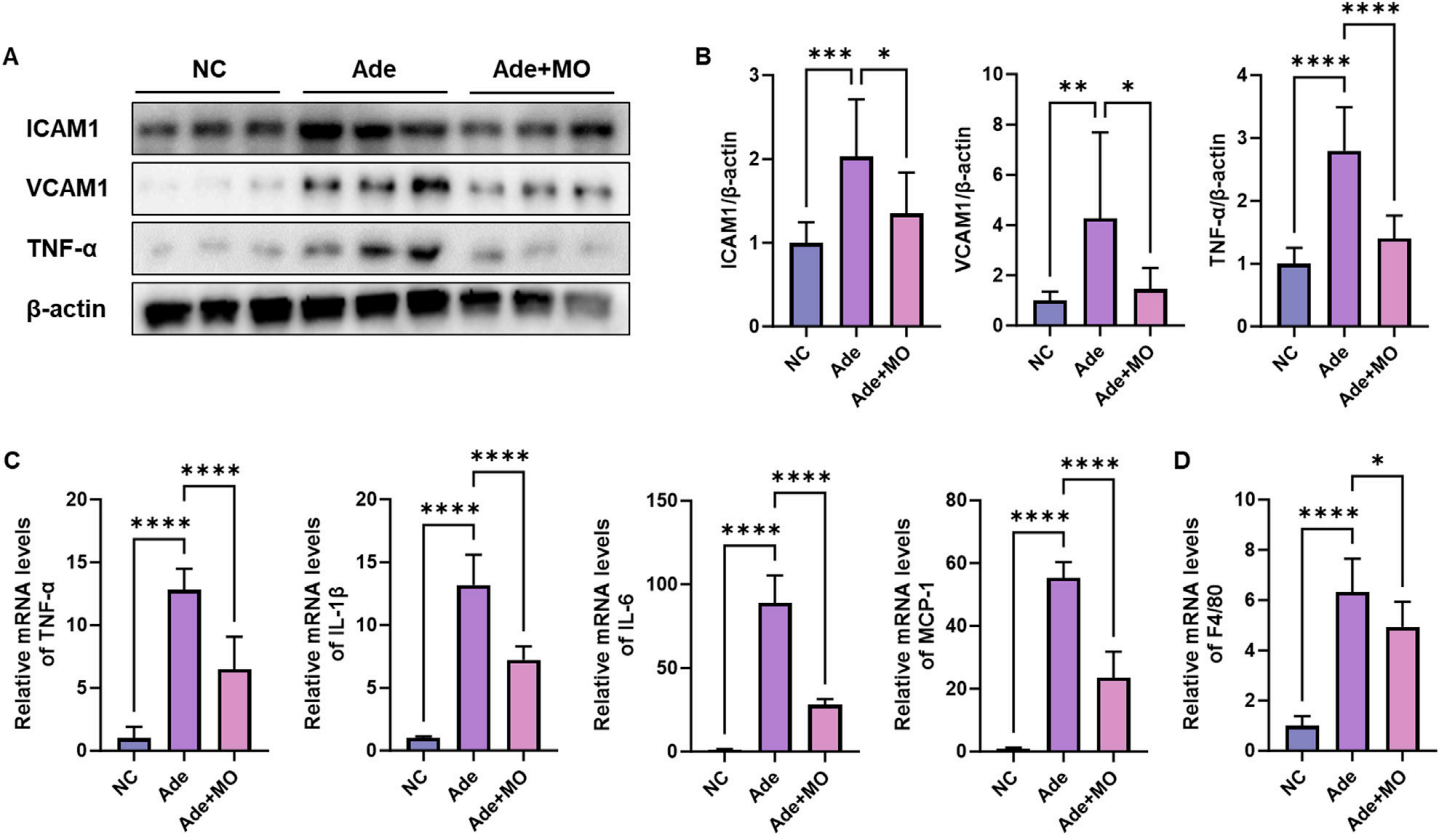
MO suppresses adenine-induced inflammation in the renal tissues. **(A)** Western blot images related to inflammation in the renal tissues. **(B)** Protein expression fold changes in β-actin. **(C)** mRNA expression-related inflammation fold changes in GAPDH. **(D)** mRNA expression of F4/80 fold changes in GAPDH. Results are presented as mean ± standard error of the mean. The statistical significance indicated as *P < 0.05, **P < 0.01, ***P < 0.001, and ****P < 0.001, and ns means no significance.

Increased immune cytokine levels also activate the renal inflammatory response, thereby accelerating the damage to the renal cells and perpetuating the inflammatory environment (Huang et al., 2023). Hence, inflammatory biomarkers were evaluated to examine the anti-inflammatory effects of MO (Figure 2). The inflammatory protein expression levels of ICAM1, VCAM1, and TNF-α, the mRNA expression levels of TNF-α, IL-1β, IL-6, and MCP-1, and the macrophagic mRNA expression level of F4/80 were increased, as compared with those in the NC group. Nevertheless, MO administration significantly suppressed the renal inflammatory response.

### 3.3 Fibrosis in the renal tissues

**FIGURE 3:**
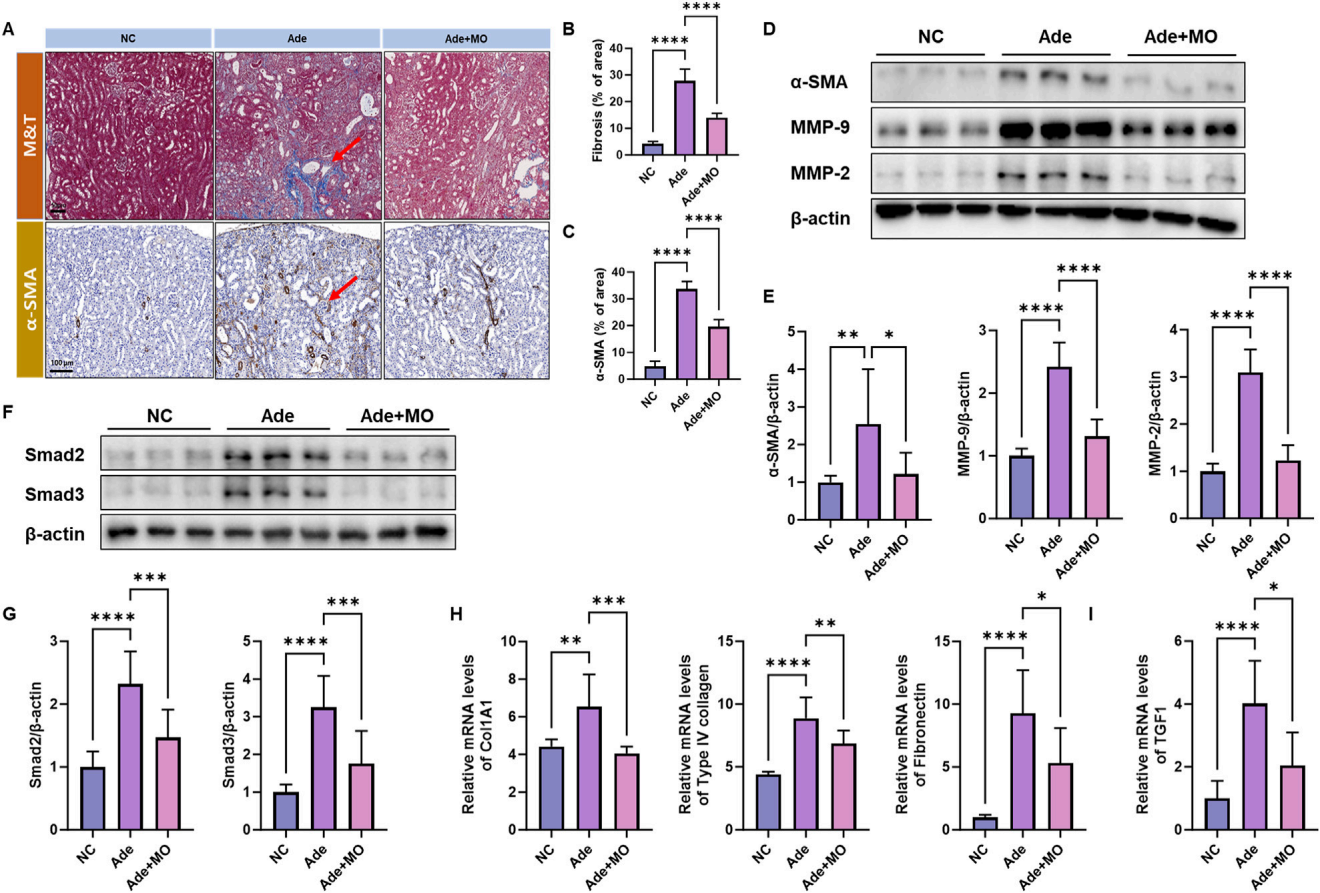
MO suppresses adenine-induced fibrosis in the renal tissues. **(A)** Renal tissues stained with Masson’s trichrome (upper panel) and alpha-smooth muscle actin (α-SMA) (lower panel). **(B)** Fibrosis ratio in Masson’s trichrome staining. **(C)** α-SMA ratio. **(D)** Western blot images related to fibrosis in the renal tissues. **(E)** Protein expression fold changes in β-actin. **(F)** Western blot images related to fibrosis in the renal tissues. **(G)** Protein expression fold changes in β-actin. **(H)** mRNA expression-related fibrosis fold changes in GAPDH. **(I)** mRNA expression-related fibrosis fold changes in GAPDH. Results are presented as mean ± standard error of the mean. The statistical significance indicated as *P < 0.05, **P < 0.01, ***P < 0.001, and ****P < 0.001, and ns means no significance.

Fibrosis is a major pathological feature of CKD and is a process that progressively impairs the renal function via excessive ECM accumulation within the renal tissues (Bülow and Boor, 2019). Thus, fibrotic biomarkers were estimated to confirm the anti-fibrotic effect of MO (Figure 3). In the CKD model, a significant increase in collagen, collagen fibers, fibrin, muscles, and erythrocytes and an increase in the Masson’s trichrome stain and expression of αSMA were observed (Figure 3A). However, MO intake inhibited these changes. Additionally, the fibrotic protein expression levels of αSMA, MMP-9, and MMP-2 and the mRNA expression levels of Col1A1, type Ⅳ collagen, and fibronectin were increased, as compared with those in the NC group (Figures 3B–D). The fibrotic mRNA expression levels of TGF1 and the protein expression levels of Smad2 and Smad3 were increased, as compared with those in the NC group (Figures 3E–G). However, MO intake significantly inhibited renal fibrosis.

### 3.4 Senescence in the renal tissues

**FIGURE 4:**
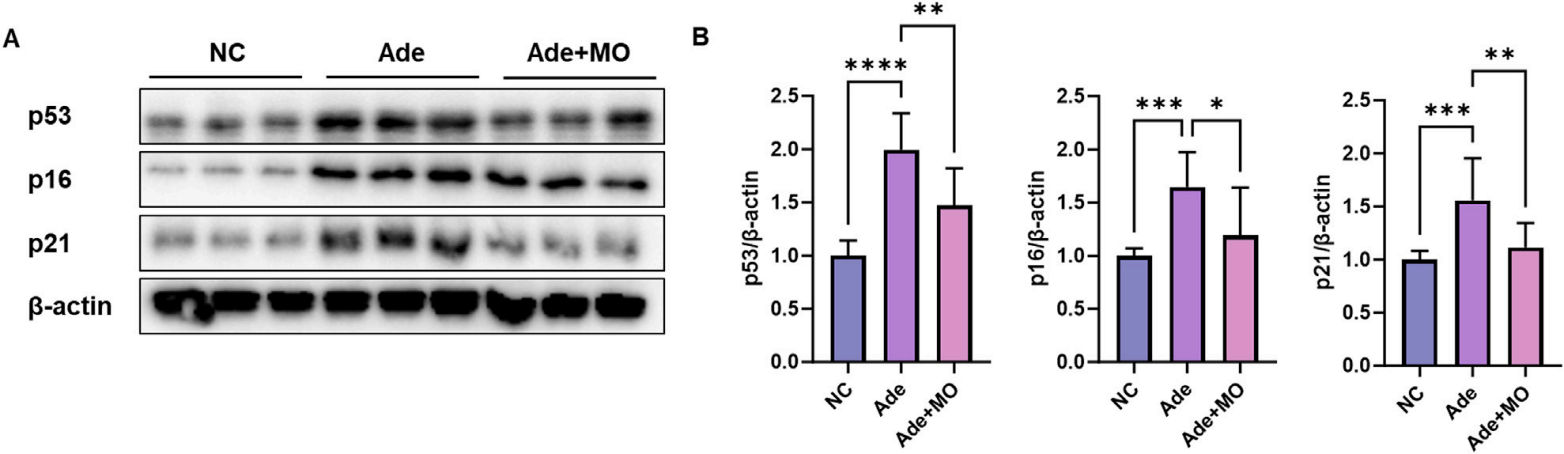
MO suppresses adenine-induced senescence in the renal tissues. **(A)** Western blot images related to senescence in the renal tissues. **(B)** Protein expression fold changes in β-actin. Results are presented as mean ± standard error of the mean. The statistical significance indicated as *P < 0.05, **P < 0.01, ***P < 0.001, and ****P < 0.001, and ns means no significance.

Senescent cells have been observed to accumulate in the renal tissues of patients with CKD; these senescent cells are not normally removed and secrete inflammatory substances that cause inflammation and aggravate damage to the surrounding tissues (Sturmlechner et al., 2017). Therefore, senescence biomarkers were estimated to confirm the anti-senescence effect of MO (Figure 4). The inflammatory protein expression levels of p53, p16, and p21 were increased, as compared with those in the NC group. However, MO intake significantly suppressed renal senescence.

### 3.5 Ferroptotic signal in the renal tissues

**FIGURE 5:**
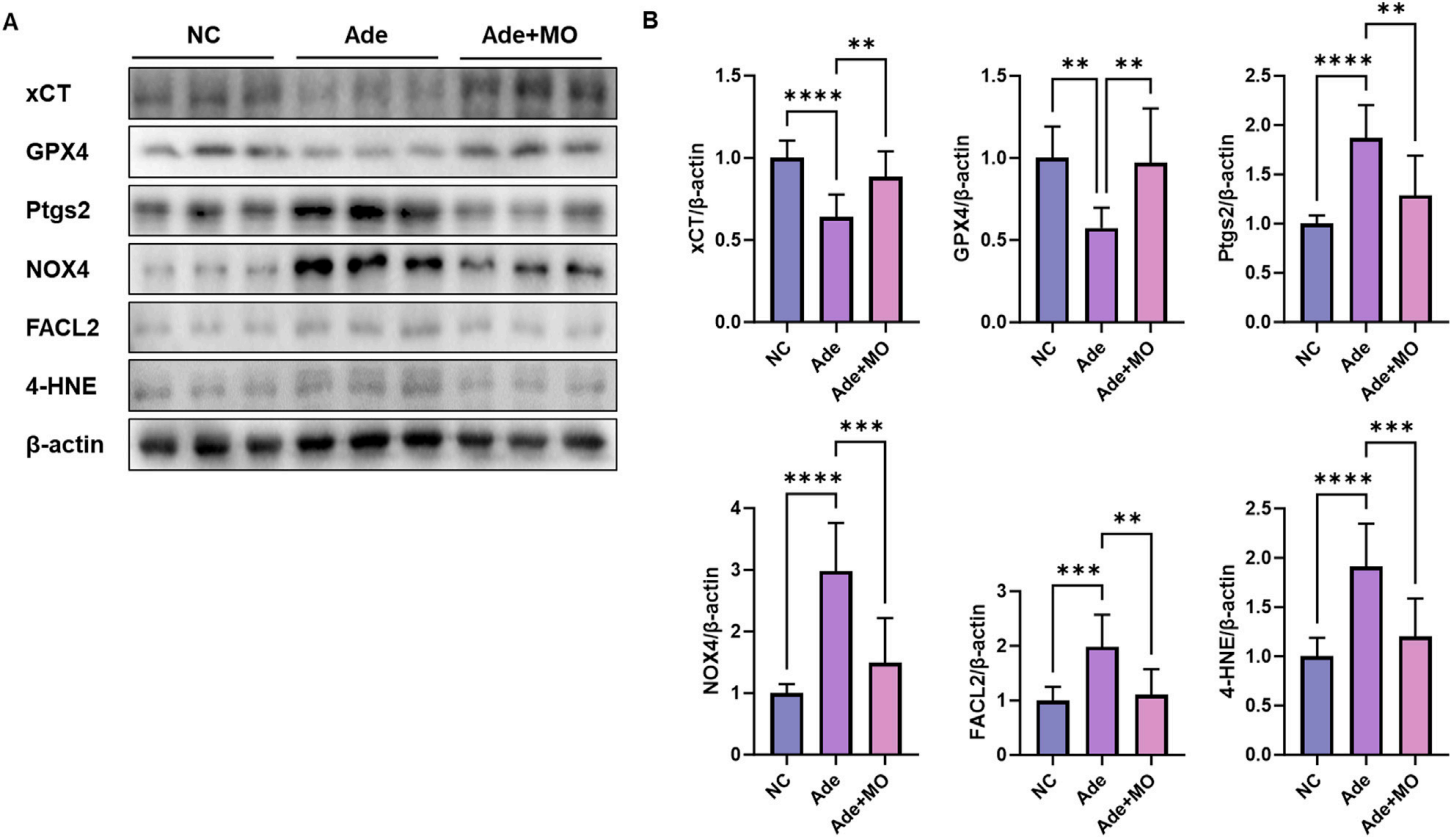
MO suppresses adenine-induced ferroptosis in the renal tissues. **(A)** Western blot images related to ferroptosis in the renal tissues. **(B)** Protein expression fold changes in β-actin. Results are presented as mean ± standard error of the mean. The statistical significance indicated as *P < 0.05, **P < 0.01, ***P < 0.001, and ****P < 0.001, and ns means no significance.

Excessive iron accumulation accelerates lipid peroxidation through free radical reactions, causing serious damage to the structure and function of renal tissue. These processes act as major mechanisms causing the pathological progression of CKD and impairs the survival and homeostasis of renal cells (Zhang and Li, 2022). Accordingly, the ferroptotic indicators were evaluated to assess the anti-ferroptotic effect of MO (Figure 5). When compared with those in the NC group, the ferroptotic protein expression levels of xCT and GPX4 were decreased, whereas the ferroptotic protein expression levels of Ptgs2, NADPH oxidase 4 (NOX4), and human palmitoyl-CoA ligase (FACL2), and 4-HNE were increased. However, MO intake significantly inhibited renal ferroptosis.

### 3.6 Ferritinophagic signal in the renal tissues

**FIGURE 6:**
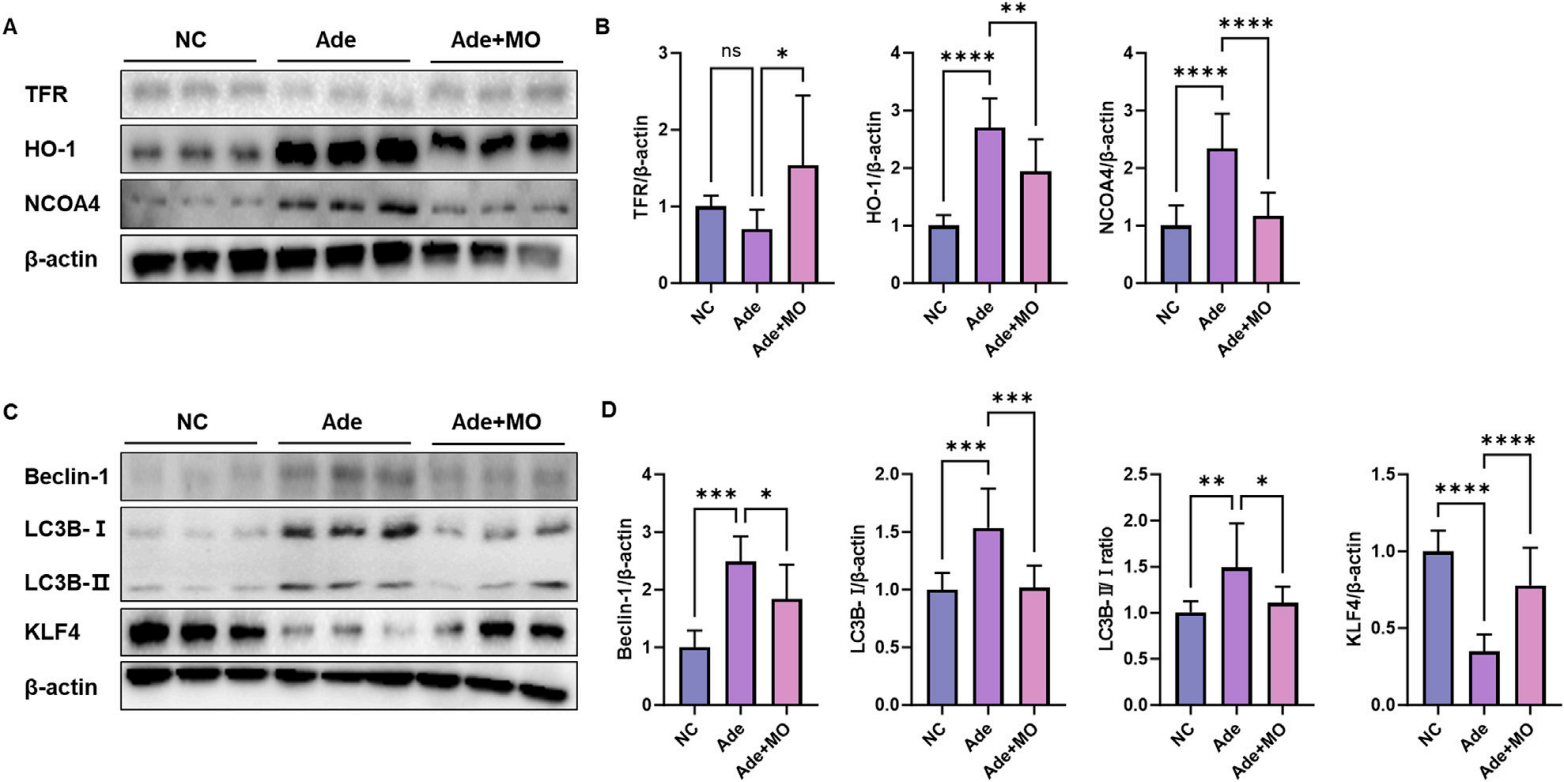
MO suppresses adenine-induced ferritinophagy in the renal tissues. **(A)** Western blot images related to iron metabolism indicators in the renal tissues. **(B)** Protein expression fold changes in β-actin. **(C)** Western blot images related to ferritinophagy in the renal tissues. **(D)** Protein expression fold changes in β-actin. Results are presented as mean ± standard error of the mean. The statistical significance indicated as *P < 0.05, **P < 0.01, ***P < 0.001, and ****P < 0.001, and ns means no significance.

In the CKD model, excess iron accumulation promotes renal ferritinophagy, which induces cell membrane damage. Continuous iron accumulation promotes free radical reactions, causing cells to proceed to the iron-dependent ferritinophagic pathway (Jomova and Valko, 2011). Accordingly, iron accumulation biomarkers were evaluated to assess the anti-ferritinophagic effect of MO (Figure 6). When compared with those in the NC group, iron metabolism regulatory protein expression level of TFR was decreased, whereas the protein expression levels of HO-1 and NCOA4 were increased. However, MO intake significantly inhibited renal iron-related iron metabolism regulatory indicators (Figures 6A,B). In addition, the ferritinophagic protein expression level of KLF4 was decreased, whereas the ferritinophagic protein expression levels of Beclin-1 and LC3B 1/2 were increased. However, MO intake significantly inhibited renal iron-related ferritinophagy.

## 4 Discussion

CKD has recently attracted attention as one of the main pathological mechanisms of ferroptosis, and research on its pathogenesis is being actively conducted. Ferroptosis causes cell damage via excessive iron ion accumulation and lipid peroxidation and alters the function and state of renal tissue (Zhang and Li, 2022). This process leads to an increase in oxidative stress and a decrease in antioxidants, including glutathione and cysteine, which induce oxidative stress and inflammatory responses in the renal tissues (Podkowińska and Formanowicz, 2020). Inflammation acts as a factor that accelerates CKD progression by promoting renal tissue fibrosis (Eddy, 2005). Ferroptosis-induced inflammation and fibrosis can cause damage and structural changes in the renal tissues during the early stages of CKD by promoting abnormal proliferation and migration of cells in the kidney (Fu et al., 2025). This complex pathological process is critical in the development of CKD, worsening disease progression and deteriorating renal function. Although MO intake significantly attenuated BUN levels, serum creatinine and calcium levels remained elevated, suggesting that renal function and metabolic homeostasis may not have been fully restored. Creatinine, a more stable indicator of renal function, often reflects more persistent structural damage. Therefore, the modest changes observed in creatinine levels may represent a partial protective effect rather than a full functional recovery. Similarly, the unchanged calcium levels may reflect persistent chronic kidney disease-related metabolic dysregulation. These results suggest that MO contributes to renal protection during the progression of renal damage rather than acting as a reparative agent after damage is established.

The inflammatory response is an important mechanism of disease progression, with various inflammatory factors contributing to renal tissue damage (Podkowińska and Formanowicz, 2020). As CKD progresses, renal cell damage and increased oxidative stress increase the expression of inflammatory cytokines such as TNF-α and ILs, which induce an inflammatory response (Andrade-Oliveira et al., 2019). These cytokines promote the expression of cell adhesion molecules such as ICAM1 and VCAM1, which allow inflammatory cells to migrate to the site of damage, causing monocytes and macrophages to infiltrate the damaged renal tissues (Meager, 1999). In particular, F4/80 is a marker of macrophages, which accumulate in the renal tissues in CKD, aggravating chronic inflammation (Cao et al., 2015). Macrophages and monocytes induced at the site of inflammation are infiltrated by chemokines, such as MCP-1, and the secretion of inflammatory cytokines is further activated (Yoshimura, 2017). This inflammatory response ultimately induces fibrosis and the hardening of renal tissues, increasing ECM accumulation (Wynn, 2008). MO suppresses the expression of inflammatory cytokines, such as TNF-α and IL-6, and the tissue infiltration of inflammatory cells by reducing the expression of ICAM1 and VCAM1 and preventing chronic inflammation from causing tissue damage. (Figure 2). MO not only suppresses the expression of inflammatory cytokines such as TNF-α and IL-6 but also blocks the activation of the inflammatory response by regulating the NF-κB pathway (Kim M. R. et al, 2024). Additionally, MO effectively inhibits the tissue infiltration of inflammatory cells and is thought to interfere with the migration of monocytes and macrophages into the damaged renal tissues by reducing the expression of ICAM1 and VCAM1. In particular, MO alleviates chronic inflammation by reducing the influx of inflammatory cells into the inflammatory site by suppressing the expression of chemokines, such as MCP-1, and reducing the accumulation of macrophages labeled with F4/80. Furthermore, MO suppresses ECM accumulation and fibrosis, thereby preventing the hardening and functional decline of renal tissues caused by the inflammatory response. These complex actions suggest that MO plays an important role in improving the inflammatory pathogenesis of CKD and protecting the structure and function of renal tissues. The dosage of 100 mg/kg/day was selected based on preliminary *in vivo* studies and previous reports (Kim Y. et al., 2024). However, this study did not investigate dose-response effects, and future studies should explore whether different doses yield varying levels of renal protection.

As CKD progresses, fibrosis occurs in the renal tissues and ECM components accumulate excessively (Eddy, 2005). Fibrosis, one of the main causes of renal function decline, progresses continuously owing to the interaction between the renal cells and ECM (Liu, 2011). In CKD, TGF-β1 plays a central role in promoting fibrosis and regulating the expression of ECM synthesis genes (Ma and Meng, 2019). With increased TGF-β1 expression, the Smad2/Smad3 signaling pathway is activated, promoting the synthesis of ECM proteins such as collagen (COL1A1) and fibronectin in renal cells (Lv et al., 2018). The Smad2/Smad3 signaling pathway is activated in response to TGF-β1 stimulation, and activated Smad proteins move to the nucleus to induce the expression of genes related to ECM synthesis (Ma and Meng, 2019). This process causes excessive accumulation of collagen and fibronectin, resulting in the stiffening of renal tissues as well as the deterioration of normal filtration function (Liu, 2011). Activated smad proteins move to the nucleus and increase the expression of ECM components, such as COL1A1 and fibronectin (Flanders et al., 2004). The expression of αSMA, a fibroblast activation marker, also increases, and mesenchymal cells are converted to fibroblasts and begin to synthesize ECM components (Ding et al., 2021). When fibroblasts are activated, they excessively produce ECM components, such as collagen, which promotes fibrosis in the renal tissues (Li et al., 2022). In particular, increased expression of COL1A1 causes type I collagen, a major structural protein of the ECM, to accumulate in the renal tissues, thereby stiffening the tissues and diminishing their flexibility (Arseni et al., 2018). This interferes with normal blood flow and filtration function and is a significant cause of renal function decline (Denic et al., 2016). Furthermore, fibronectin expression increases with CKD progression, promoting cell adhesion and structural formation in the ECM, further accelerating the excessive ECM accumulation and forming fibrotic tissues that lose their normal tissue structure and function (Zhang et al., 2020a). This leads to an imbalance in the expression of MMPs, such as MMP-2 and MMP-9, which continuously regulate the degradation and remodeling of the ECM, thereby aggravating the stiffness and fibrosis of renal tissues (Zakiyanov et al., 2021). Thus, changes in the expression of TGF-β1, Smad2/Smad3, αSMA, COL1A1, collagen, and fibronectin in the CKD model reflect excessive ECM accumulation and continuous fibrosis progression, which ultimately lead to structural changes in the renal tissues and functional decline (Eddy, 2005). Therefore, the expression of these factors was measured, and they were confirmed to have improved by MO intake (Figure 3). The protective effects of MO against fibrosis are exerted through various mechanisms, including antioxidant, anti-inflammation, and TGF-β/Smad pathway-inhibitory effects (Kim M. R. et al., 2024). MO contains powerful antioxidant components such as honokiol and magnolol, which are important for suppressing oxidative stress via reduction of reactive oxygen species (ROS) and preventing fibrosis progression (Yuan et al., 2020). These components activate antioxidant defense mechanisms via the Nrf2 pathway to reduce cell damage and inhibit ECM accumulation (Dai et al., 2023). MO exhibits anti-inflammatory effects, reducing not only the expression of inflammatory cytokines such as TNF-α but also the infiltration of inflammatory cells by suppressing the expression of ICAM1 and VCAM1, thereby alleviating fibrosis progression. MO can prevent tissue stiffness and fibrosis by inhibiting the TGF-β/Smad pathway, thereby blocking excessive fibroblast activation and synthesis of ECM proteins such as collagen and fibronectin. These combined actions suggest that MO exerts a protective effect against tissue damage associated with fibrosis. Several studies have demonstrated nephroprotective effects of various plant-derived compounds, such as curcumin, resveratrol, and astragaloside IV, in CKD models through anti-inflammatory and antifibrotic pathways (Gowd et al., 2020; Trujillo et al., 2013; Zhang et al., 2020b). These findings further support the potential role of MO as a functional bioactive agent for renal protection. Therefore, MO may be an excellent bioactive plant material for suppressing fibrosis in adenine-induced CKD.

The main causes of senescence in CKD models are oxidative stress, inflammation, and DNA damage (Wang et al., 2017). Increased ROS levels in CKD aggravate oxidative stress and result in damage to the DNA, proteins, and lipids in the renal tissues (Ling and Kuo, 2018). This causes cells to enter a senescent state, and the expression of cell cycle inhibitory proteins, such as p53, p16, and p21, increases (Melk et al., 2004). In patients with CKD, changes in the expression of p53, p16, p21, and PAI-1 are closely related, accelerating senescence, inflammation, and fibrosis of renal cells in a complex manner (Xu et al., 2020). p53 responds to DNA damage by increasing the expression of p21, a cell-cycle inhibitory protein that induces damaged cells to enter a senescent state (Mijit et al., 2020). The accumulated senescent cells release inflammatory cytokines, causing chronic inflammation and simultaneously increasing the expression of factors that promote ECM accumulation (Melk et al., 2004). In addition, p16, together with p53 and p21, inhibits the proliferation of damaged renal cells and induces the accumulation of senescent cells, and inflammatory cytokines secreted by senescent cells accelerate the inflammation and fibrosis of renal tissues (Xu et al., 2020). As a result, the interaction of p53, p16, and p21 causes a vicious cycle of inflammation, ECM accumulation, and fibrosis and acts as a pathological mechanism that promotes renal damage and functional decline (Ma and Fogo, 2009). Therefore, senescence biomarkers were measured, and MO intake significantly improved these levels (Figure 4). MO exerts protective effects via various mechanisms that inhibit cell senescence. MO has antioxidant properties that reduce ROS and alleviate oxidative stress, contributing to the reduction of oxidative damage, which is one of the main causes of cell senescence. In addition, *Magnolia* family suppress cell senescence caused by chronic inflammation by regulating the inflammatory responses (Lee et al., 2011). In particular, MO prevents cells from entering a senescent state by regulating the expression of the cell cycle regulatory proteins p53 and p21, thereby helping maintain physiological cellular functions and reduce the damage caused by senescence (Kim Y. et al., 2024). These mechanisms play important roles in protecting MOs against cell senescence.

Ferroptosis is an iron-dependent cell death mechanism in which oxidative stress and lipid peroxidation play key roles (Zhang and Li, 2022). The expression of xCT and GPX4 is suppressed, which weakens the antioxidant defense and results in the accumulation of 4-HNE, a lipid peroxidation product that damages the stability of cell membranes and promotes ferroptosis (Yan et al., 2021). In addition, TFR expression, which regulates iron metabolism, is decreased in CKD, overstimulating intracellular HO-1 expression, which continuously causes an increase in intracellular iron ions, accelerating ferroptosis (Yan et al., 2021). Furthermore, we also analyzed the expression of NOX4 and FACL2, which are important mediators of lipid peroxidation and ferroptosis. NOX4 promotes the generation of ROS, contributing to oxidative stress in renal tissues, whereas ACSL4 facilitates the incorporation of polyunsaturated fatty acids into phospholipids, increasing susceptibility to lipid peroxidation (Zhang and Li, 2022). The modulation of these factors by MO provides additional mechanistic evidence that MO attenuates ferroptotic cell death by suppressing lipid peroxidation. Intracellular accumulation of free iron ions promotes the progress of ferritinophagy (Xie et al., 2024). Ferritinophagy is one of the main ferroptotic causes of kidney damage and associated with increased inflammation, apoptosis, and oxidative stress (Latunde-Dada, 2017). The ferritinophagy regulators Beclin-1 and LC3B are dysfunctional, and damaged cell organelles cannot be removed, resulting in accumulated cell damage (Qin et al., 2021). These factors act as pathological mechanisms that accelerate renal function decline by decreasing the expression of KLF4, a factor that regulates ferritinophagy and suppresses inflammatory responses, thereby exacerbating inflammation and fibrosis and increasing ECM accumulation (Delgado-Valero et al., 2021). These changes in expression reflect a pathological flow in which ferroptosis-derived ferritinophagy accelerates cell damage, inflammation, and fibrosis progression, and mechanisms to inhibit ferroptosis may be important therapeutic targets for the management of CKD (Huang et al., 2023). Similar to these pathological pathways, ferroptotic biomarkers were imbalanced in the renal tissues (Figures 5, 6). However, MO intake significantly ameliorated ferroptotic signals in the renal tissues. *Magnolia* has an excellent antioxidant effect that reduces oxidative stress, and in particular, it plays a role in preventing the peroxidation of cell membrane lipids by inhibiting ROS production (Chen et al., 2001). MO has been reported to exert protective effects against glutamate-induced neurotoxicity by regulating the GSH system, including GSH levels, GSH reductase, the inhibition of GSH superoxide dismutase production, and the regulation of Ca^2+^ levels (Jung et al., 2018). In particular, 3,5-di-caffeoylquinic acid, a MO component, suppresses mitochondrial dysfunction and ferroptosis by regulating the expression of GPX4, ACSL4, and xCT in colorectal cancer cells (Wang et al., 2023). These mechanisms help MO maintain the stability of the cell membrane and increase their resistance to oxidative damage, thereby preventing the damage caused by ferroptosis. In addition, excessive ferritinophagy activation was observed in CKD tissues by increasing the expression of Beclin-1 and LC3B 1/2, suggesting that ferritinophagy is linked to cell damage and inflammatory responses (Figure 6). However, MO administration regulated the ferritinophagy protein expression levels of Beclin-1, LC3B 1/2, and KLF4, indicating that MO contributes to the restoration of cellular homeostasis by suppressing ferritinophagy overactivity. In addition, MO increased the expression of KLF4, an important transcription factor that regulates ferritinophagy and inflammatory responses, thereby regulating ferritinophagy to an appropriate level and suppressing cell damage and inflammation caused by excessive ferritinophagy. In addition, MO can be utilized as a considerable material to regulate the ferritinophagy mechanism to an appropriate level and suppress cell damage and inflammation caused by excessive ferritinophagy. Although current CKD treatments such as sodium glucose co-transporter two inhibitors and renin-angiotensin-aldosterone system blockers provide beneficial effects by modulating intraglomerular hemodynamics and systemic metabolic regulation, they may not fully address oxidative stress, ferroptosis, or tissue fibrosis. MO exerts protective effects by modulating these alternative pathways, suggesting its potential as a complementary therapeutic agent. Future studies combining MO with standard CKD medications may help evaluate its synergistic or additive effects in clinical settings. Although we evaluated markers of ferroptosis such as xCT, GPX4, Ptgs2, and 4-HNE, this study did not directly evaluate lipid peroxidation markers, such as lipid ROS or MDA, and iron indicators, such as serum iron, ferritin, or transferrin saturation. Further studies are needed to provide mechanistic evidence for its involvement in ferroptosis.

### 4.1 Conclusion

**FIGURE 7:**
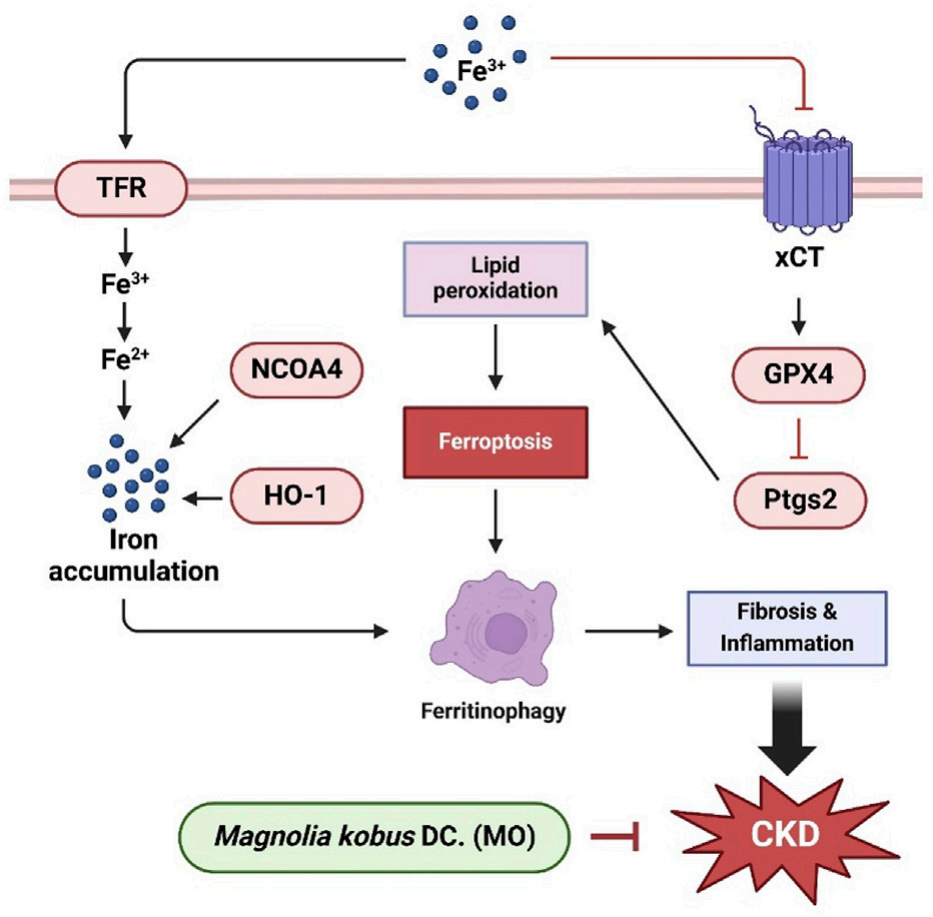
Schematic illustration of the protective effect of MO on adenine-induced renal dysfunction in mice kidney tissue.

The findings of the current study confirm that MO exerts renoprotective effects by regulating fibrosis, aging, ferroptosis, and inflammation, which are the major pathological mechanisms in CKD (Figure 7). MO plays an important role in preventing renal dysfunction by preventing renal morphological change. MO suppresses CKD-induced inflammation, inhibits renal fibrosis, alleviates renal senescence, and prevents cell membrane damage. These results suggest that MO may be utilized as a functional food or medicinal plant material for regulating key pathological pathways associated with CKD, although its therapeutic efficacy requires further validation using clinically relevant endpoints. Further studies should be conducted to elucidate the bioactive components and mechanisms of action of MO. However, few studies have revealed the renal protective effects and evaluation of mechanism of individual bioactive materials in MO. Therefore, future studies should investigate the physiological effects of compounds in MO on CKD-induced renal dysfunction, which would further clarify the potential of MO as a functional food or medicinal material for the prevention and management of CKD. In addition, since MO administration was initiated simultaneously with the induction of adenine-induced CKD, the effects observed in this study primarily indicate the renal protective potential of MO in the early stages of renal damage. While MO treatment significantly modulated ferroptosis- and ferritinophagy-related markers, these findings correlate. Definitive causal relationships cannot be established without genetic or pharmacological intervention targeting these pathways. Therefore, further studies are needed to investigate whether MO can exert reparative effects after fully established CKD. Furthermore, as this study utilized a crude ethanolic extract of MO, detailed data on extraction yield, purity of bioactive compounds, and batch-to-batch variability were not assessed. Future investigations should focus on standardizing the extract, profiling major active constituents, and ensuring reproducibility to support its practical application as a functional material or therapeutic agent.

## Data Availability

The original contributions presented in the study are included in the article/Supplementary Material, further inquiries can be directed to the corresponding author.
